# Initiation and Dose of Methadone Monotherapy vs Combination Therapy, 2015 to 2023

**DOI:** 10.1001/jamanetworkopen.2025.27290

**Published:** 2025-08-15

**Authors:** Ria Garg, Jin Luo, Nikki Bozinoff, Beth Sproule, Tony Antoniou, Jennifer Wyman, Charlotte Munro, Tara Gomes

**Affiliations:** 1 ICES, Toronto, Ontario, Canada; 2 Leslie Dan Faculty of Pharmacy, University of Toronto, Toronto, Ontario, Canada; 3 Campbell Family Mental Health Research Institute, Centre for Addiction and Mental Health, Toronto, Ontario, Canada; 4 Department of Family and Community Medicine, University of Toronto, Toronto, Ontario, Canada; 5 Centre for Addiction and Mental Health, Toronto, Ontario, Canada; 6 Department of Psychiatry, University of Toronto, Toronto, Ontario, Canada; 7 Li Ka Shing Knowledge Institute, St Michael’s Hospital, Toronto, Ontario, Canada; 8 Department of Family and Community Medicine, St Michael’s Hospital, Toronto, Ontario, Canada; 9 Division of Addiction Medicine, Women’s College Hospital, Toronto, Ontario, Canada; 10 Ontario Drug Policy Research Network Lived Experience Advisory Group, Toronto, Ontario, Canada; 11 Institute of Health Policy, Management and Evaluation, University of Toronto, Toronto, Ontario, Canada

## Abstract

**Importance:**

The increased potency of the fentanyl-dominated drug supply has precipitated a shift in patient-reported opioid tolerance and methadone prescribing guidance. However, adoption of new methadone prescribing approaches remains unknown.

**Objective:**

To examine methadone initiation trends within the context of changing prescribing guidance and an increasingly volatile unregulated drug supply.

**Design, Setting, and Participants:**

This repeated cross-sectional study was performed among a cohort of methadone initiations in Ontario, Canada, between January 2015 and July 2023.

**Exposures:**

Release of new methadone prescribing guidance.

**Main Outcomes and Measures:**

The primary outcomes were monthly rates (per 100 000 Ontario population) of methadone initiated as monotherapy vs in combination with slow-release oral morphine and trends for the maximum methadone dose dispensed on treatment days 1, 2 to 7, and 8 to 14. Interventional autoregressive integrated moving average models were used to examine the association of new guidance with observed trends. Secondary outcomes assessed medication use in the 2 weeks following initiation.

**Results:**

A total of 70 564 initiations of methadone monotherapy and 3069 initiations of combination therapy were identified. Most of the 35 309 unique study participants were aged 25 to 44 years (51 999 episodes [70.6%]) and male (45 212 episodes [61.4%]). The release of new methadone prescribing guidance was associated with a significant decrease in monotherapy initiations (ramp estimate, −0.27 per 100 000; 95% CI, −0.48 to −0.07 per 100 000; *P* = .01), and a parallel increase in combination therapy initiations. Starting in 2018, a shift toward attainment of higher doses was noted. This trend was accelerated following release of new guidance, which was associated with decreased initiation at doses less than 30 mg (ramp estimate, −1.57%; 95% CI, −2.87% to −0.27%; *P* = .02) and increased attainment of doses of 40 mg to less than 50 mg (ramp estimate, 0.63%; 95% CI, 0.13% to 1.13%; *P* = .01) in treatment week 1 and 60 mg or higher (ramp estimate, 0.71%; 95% CI, 0.25% to 1.17%; *P* = .002) in treatment week 2. The proportion of monotherapy episodes without a dose increase in the first 2 weeks increased significantly from 38.5% (18 390 episodes initiated before September 2020) to 45.7% (10 416 episodes) afterward. The absolute amount by which methadone doses were titrated remained generally stable over time.

**Conclusions and Relevance:**

In this repeated cross-sectional study of methadone initiations, a shift toward higher starting doses and in combination with slow-release oral morphine was noted. Provision of subsequent rapid dose titration remained limited, representing a potential missed opportunity for quicker attainment of therapeutic doses.

## Key Points

**Question:**

How have methadone prescribing practices adapted to the increasing volatility of the unregulated drug supply and evolving clinical guidance?

**Findings:**

In this repeated cross-sectional study of 70 564 initiations of methadone monotherapy and 3069 initiations of combination therapy among 35 309 unique individuals, there was a shift toward methadone initiation at 30 mg in 2018, with subsequent attainment of higher doses within 2 weeks. The release of new methadone prescribing guidance further accelerated treatment initiation at higher doses and in combination with slow-release oral morphine; however, provision of subsequent dose titration significantly declined over time.

**Meaning:**

These findings suggest that the limited provision of rapid dose titration represents a potential missed opportunity for faster attainment of therapeutic doses.

## Teaser

This cross-sectional study examines methadone initiation trends within the context of changing prescribing guidance and the increased potency of the fentanyl-dominated unregulated drug supply.

## Introduction

The North American opioid toxicity crisis has claimed more than 500 000 lives since 2016,^1,2^ with an estimated two-thirds of deaths occurring among people with opioid use disorder (OUD).^3^ North America’s potent and unpredictable fentanyl-dominated unregulated drug supply is the primary driver of this crisis, with more than 80% of opioid-related deaths attributed to fentanyl since 2018.^1,2,3^ Although methadone and buprenorphine are safe and effective opioid agonist treatments (OATs) for patients with OUD, treatment retention remains low, in part owing to the increased potency of the unregulated drug supply, and resultant elevated opioid tolerance among people with OUD.^4,5,6^ Specifically, patients are dissatisfied with traditional treatment regimens and often require much higher OAT doses compared with when the unregulated drug supply was dominated by heroin.^6,7,8^

The increasing prevalence of opioid toxicities and need for improved OAT retention have driven rapid adaptation in OAT prescribing practices, particularly for methadone.^6^ Population-level evidence suggests improved treatment retention among persons using fentanyl compared with buprenorphine-naloxone.^4,9^ However, requirements for methadone initiation at subtherapeutic doses with gradual titration—owing to substantial interpatient variability in its pharmacokinetics—complicate the timely achievement of therapeutic doses that effectively manage opioid cravings and withdrawal symptoms. It is suggested that individuals who use fentanyl are partially cross-tolerant to methadone’s sedative and respiratory suppressant effects^6^ and may initiate methadone at higher doses, with more rapid titration than previously considered appropriate.^7,10^ Therefore, in March 2021, the College of Physician and Surgeons of Ontario rescinded its methadone policy, which recommended a start low, go slow approach and prohibited physicians from initiating methadone at doses greater than 30 mg.^11^ Since then, the Ontario-based Mentoring, Education, and Clinical Tools for Addition–Partners in Health Integration (META-PHI) has released new recommendations for methadone prescribing among people who use fentanyl.^6^ Key components of the new guidance included initiation of methadone at 30 mg (the higher end of the previously allowed range), which may be increased by 10 to 15 mg every 3 to 5 days and in combination with slow-release oral morphine (SROM; used as OAT in Canada^6,12^). The extent to which new approaches to methadone prescribing were adopted across Ontario remains unknown. Therefore, we described methadone initiation trends during the first 2 weeks of treatment across Ontario, Canada, in the context of changing prescribing guidance and an increasingly volatile unregulated drug supply.

## Methods

### Study Design and Setting

We conducted a retrospective, population-based repeated cross-sectional analysis among a cohort of methadone initiations captured between January 1, 2013, and July 31, 2023, in Ontario, Canada to examine methadone prescribing trends during the first 14 days of treatment. This study was preregistered on the Open Science Framework,^13^ and adheres to Strengthening the Reporting of Observational Studies in Epidemiology (STROBE) reporting guideline.^14^

### Data Sources

We used administrative health databases housed at ICES (formerly known as the Institute for Clinical Evaluative Sciences), an independent, nonprofit research institute whose legal status under Ontario’s health information privacy law allows it to collect and analyze health care and demographic data, without consent of Ontario residents registered with the Ontario Health Insurance Plan. We used the Narcotics Monitoring System to obtain information on all controlled substances (eg, methadone or SROM) dispensed from community pharmacies regardless of payer. Unique encoded identifiers were used to link the Narcotics Monitoring System to other datasets (see eTable 1 in Supplement 1 for details on databases) and analyzed at ICES. Data used in this study are authorized under section 45 of Ontario’s Personal Health Information Protection Act and do not require review by a research ethics board.

### Cohort

Our study cohort included all incident methadone episodes identified during the study period, with index date defined as date of methadone initiation. An individual could contribute to the cohort multiple times, if they met the inclusion and exclusion criteria. Incident methadone use was defined as no recent dispense record for OAT or daily dispensed immediate release hydromorphone (eg, safer opioid supply^15^) within a predefined lookback period, which varied according to dosing frequency (see eTable 2 in Supplement 1 for drug identification numbers). A 30-day lookback period was used to define recent use of shorter-acting OAT formulations (ie, SROM, methadone, and buprenorphine-naloxone) and immediate-release hydromorphone. For longer-acting formulations, lookback periods of 210 days were used for buprenorphine implant and 42 days for extended-release buprenorphine. Additional exclusion criteria were applied on index date to ensure incident methadone use, which included hospital discharge in the prior 14 days; methadone prescribed by an out-of-province prescriber, pharmacist or dentist; days’ supply exceeding 1; and initial methadone dose greater than or equal to 60 mg. Although the majority of methadone dispensed in Ontario, Canada, is indicated for OUD, methadone may be prescribed for chronic pain. Therefore, we excluded individuals with a health care interaction for palliative care or cancer treatment or diagnosis in the year prior to index (active cancer definition provided elsewhere^16^). Finally, we excluded individuals with an invalid Ontario health card number and aged more than 105 or less than 18 years at index.

### Involvement of People With Lived Experience

The Ontario Drug Policy Research Network hosts a Lived Experience Advisory Group, composed of individuals with experience using opioids. Feedback provided by the group informed the study methods, and 1 member served as a coauthor (C.M.).

### Outcomes

Our primary outcomes included the monthly rate of methadone initiated as monotherapy vs combination therapy (ie, same day dispenses of methadone and SROM) per 100 000 Ontario population, and trends in methadone dosing during the 2 weeks of treatment. Shifts in methadone dosing were characterized by the percentage of incident methadone episodes with a maximum dose of less than 30 mg, 30 to less than 40 mg, 40 to less than 50 mg, 50 to less than 60 mg, or 60 mg or higher (where applicable) on index and during the first (days 2 to 7) and second (days 8 to 14) week of treatment, for each month of interest. Specifically, each methadone initiation included in our cohort was monitored for the first 14 days of treatment to establish medication use patterns. Secondary outcomes included time to first methadone dose titration (ie, first dispense date where dose dispensed is greater than index date), maximum change in methadone dose (ie, maximum dose dispensed in week 2 minus index), and provision of combination therapy to monotherapy initiators. Baseline patient and prescriber characteristics were summarized on index date (see eTable 3 in Supplement 1 for covariate definitions).

### Statistical Analysis

Descriptive statistics were used to report baseline characteristics and medication use patterns for all incident methadone episodes, reported overall and stratified by medication (monotherapy initiated before September 2020, monotherapy initiated on or after September 2020, and combination therapy) and dose (<30 mg, 30 to <40 mg, 40 to <50 mg, or 50 to <60 mg) dispensed on index date. Monotherapy episodes were stratified by initiation date to enable comparison with combination therapy episodes initiated during the same period, as 99% of combination therapy initiations occurred from September 2020 onward. We used absolute standardized differences to compare baseline characteristics and medication use patterns, with differences greater than 0.1 considered meaningful.^17^ Interventional autoregressive integrated moving average (ARIMA) models were used to assess the association between methadone prescribing guidance and shifts in methadone monotherapy initiation rates and dosing patterns.^18^ We included September 2020 and March 2021 as intervention points using a ramp transfer function in all ARIMA models. These dates correspond with the presentation of methadone prescribing recommendations at the META-PHI annual conference and release of preliminary guidance by META-PHI plus College of Physician and Surgeons of Ontario’s rescission of methadone standards, respectively. Although META-PHI’s methadone prescribing guidance was officially published in June 2021, the intervention was modeled at the time of the preliminary release in March 2021, given its temporal proximity to the official release and anticipated influence on clinical practice. A pulse transfer function was included between March to May 2020 to determine the association between the declaration of emergency for the SARS-CoV-2 pandemic and monotherapy initiation rates. We based this decision on visual inspection of the trend and evidence of short-term health care access disruptions during this period.^19^ Differencing was required in all models to achieve stationarity. Seasonal differencing was also considered on the basis of visual inspection of the trend and autocorrelation plots. We used the augmented Dickey-Fuller test to assess stationarity and examined residual autocorrelation, partial autocorrelation, and inverse autocorrelation correlograms for model parameter selection. Akaike information criteria (AIC), bayesian information criteria, autocorrelation plots, and Ljung-Box χ^2^ test for white noise were used to select the final models. Data were analyzed using SAS statistical software version 9.4 (SAS Institute).

## Results

Between January 2015 and July 2023, we identified 73 633 incident methadone episodes among 35 309 unique individuals, of which the majority of whom were aged 25 to 44 years (51 999 episodes [70.6%]) and male (45 212 episodes [61.4%]) (Table 1; see eFigure 1 in Supplement 1 for cohort exclusion flow diagram). We observed significantly higher measures of OUD severity among combination therapy vs monotherapy episodes initiated on or after September 2020, including a higher prevalence of prior opioid toxicity (575 episodes [18.7%] vs 3303 episodes [14.5%]) and prior use of methadone (1979 episodes [64.5%] vs 10 863 episodes [47.7%]) and SROM (1430 episodes [46.6%] vs 895 episodes [3.9%]). When comparing monotherapy episodes initiated before September 2020 vs on or after September 2020, prior OAT use (20 196 episodes [42.3]% vs 12 394 episodes [54.4%]) and opioid toxicity (3619 episodes [7.6%] vs 3303 episodes [14.5%]) became significantly more common over time. Prescriber characteristics were generally consistent across stratifications, with 76.7% of episodes (56 470 episodes) initiated by a physician specialized in family medicine and 71.0% of episodes (52 416 episodes) initiated by a high-volume OAT prescriber.

**Table 1.** Baseline Characteristics, Reported Overall and Stratified by Medications Dispensed on Index Date

Characteristic	Episodes, No. (%)			
	Overall	Methadone before September 2020	Methadone September 2020 or later	Combination therapy (methadone and SROM)
Continuous use periods, No.	73 633	47 779	22 785	3069
Unique individuals, No.	35 309	27 101	14 097	2057
Age, median (IQR), y	33 (28-41)	33 (27-40)^a^	34 (29-41)	35 (29-41)
Age category, y				
18-24	8547 (11.6)	6468 (13.5)^a^	1867 (8.2)	212 (6.9)
25-44	51 999 (70.6)	32 886 (68.8)	16 709 (73.3)	2404 (78.3)^b^
45-64	12 537 (17.0)	8115 (17.0)	3970-3975	445-450
≥65	550 (0.7)	310 (0.6)	240-235	≤5^b^
Sex				
Female	28 421 (38.6)	17 987 (37.6)	9015 (39.6)	1419 (46.2)^b^
Male	45 212 (61.4)	29 792 (62.4)	13 770 (60.4)	1650 (53.8)^b^
Rurality of residence				
Urban	63 710 (86.5)	41 459 (86.8)	19 550 (85.8)	2701 (88.0)
Rural	8823 (12.0)	5773 (12.1)	2806 (12.3)	244 (8.0)^b^
Missing	1100 (1.5)	547 (1.1)	429 (1.9)	124 (4.0)^b^
Income quintile				
1 (Lowest)	30 023 (40.8)	19 131 (40.0)	9446 (41.5)	1446 (47.1)^b^
2	16 868 (22.9)	10 955 (22.9)	5287 (23.2)	626 (20.4)
3	11 521 (15.6)	7503 (15.7)	3618 (15.9)	400 (13.0)
4	8010 (10.9)	5454 (11.4)	2272 (10.0)	284 (9.3)
5 (Highest)	6021 (8.2)	4109 (8.6)	1724 (7.6)	188 (6.1)
Missing	1190 (1.6)	627 (1.3)	438 (1.9)	125 (4.1)^b^
Prescribed medications 180 d before index	34 765 (47.2)	20 196 (42.3)^a^	12 394 (54.4)	2175 (70.9)^b^
Methadone	29 853 (40.5)	17 011 (35.6)^a^	10 863 (47.7)	1979 (64.5)^b^
Buprenorphine-naloxone	7167 (9.7)	4267 (8.9)	2538 (11.1)	362 (11.8)
Extended-release buprenorphine	198 (0.3)	≤5	158 (0.7)	35-40
SROM	2476 (3.4)	151 (0.3)^a^	895 (3.9)	1430 (46.6)^b^
Kadian	2424 (3.3)	122 (0.3)^a^	875 (3.8)	1427 (46.5)^b^
M-Eslon	70 (0.1)	31 (0.1)	29 (0.1)	10 (0.3)
Immediate-release hydromorphone	1143 (1.6)	846 (1.8)	245 (1.1)	52 (1.7)
Occurrence of opioid toxicity in 365 d before index date	7497 (10.2)	3619 (7.6)^a^	3303 (14.5)	575 (18.7)^b^
Prescriber identified on index date				
Physician	72 934 (99.1)	47 598 (99.6)	22 362 (98.1)	2974 (96.9)
Family practitioner	56 470 (76.7)	36 975 (77.4)	17 042 (74.8)	2453 (79.9)^b^
Emergency medicine	7764 (10.5)	5142 (10.8)	2334 (10.2)	288 (9.4)
Internal medicine	62 (0.1)	52 (0.1)	5-10	≤5
Psychiatry	3903 (5.3)	2706 (5.7)	1161 (5.1)	36 (1.2)^b^
Other	5434 (7.4)	2904 (6.1)^a^	2242 (9.8)	288 (9.4)
Nurse	699 (0.9)	181 (0.4)^a^	423 (1.9)	95 (3.1)
OAT prescribing volume for prescriber identified on index date				
Low (50th percentile)	2758 (3.7)	1857 (3.9)	774 (3.4)	127 (4.1)
Moderate (51st to 80th percentile)	18 459 (25.1)	12 760 (26.7)^a^	4861 (21.3)	838 (27.3)^b^
High (top 20th percentile)	52 416 (71.2)	33 162 (69.4)^a^	17 150 (75.3)	2104 (68.6)^b^

Abbreviation: OAT, opioid agonist treatment; SROM, slow-release oral morphine.

^a^ Indicates observation of a meaningful difference based on a standardized difference greater than 0.10 between those who initiated methadone prior to September 2020 versus in September 2020 or later.

^b^ Indicates observation of a meaningful difference based on a standardized difference greater than 0.10 between those who initiated methadone monotherapy in September 2020 or later versus combination therapy.

**Figure 1.**
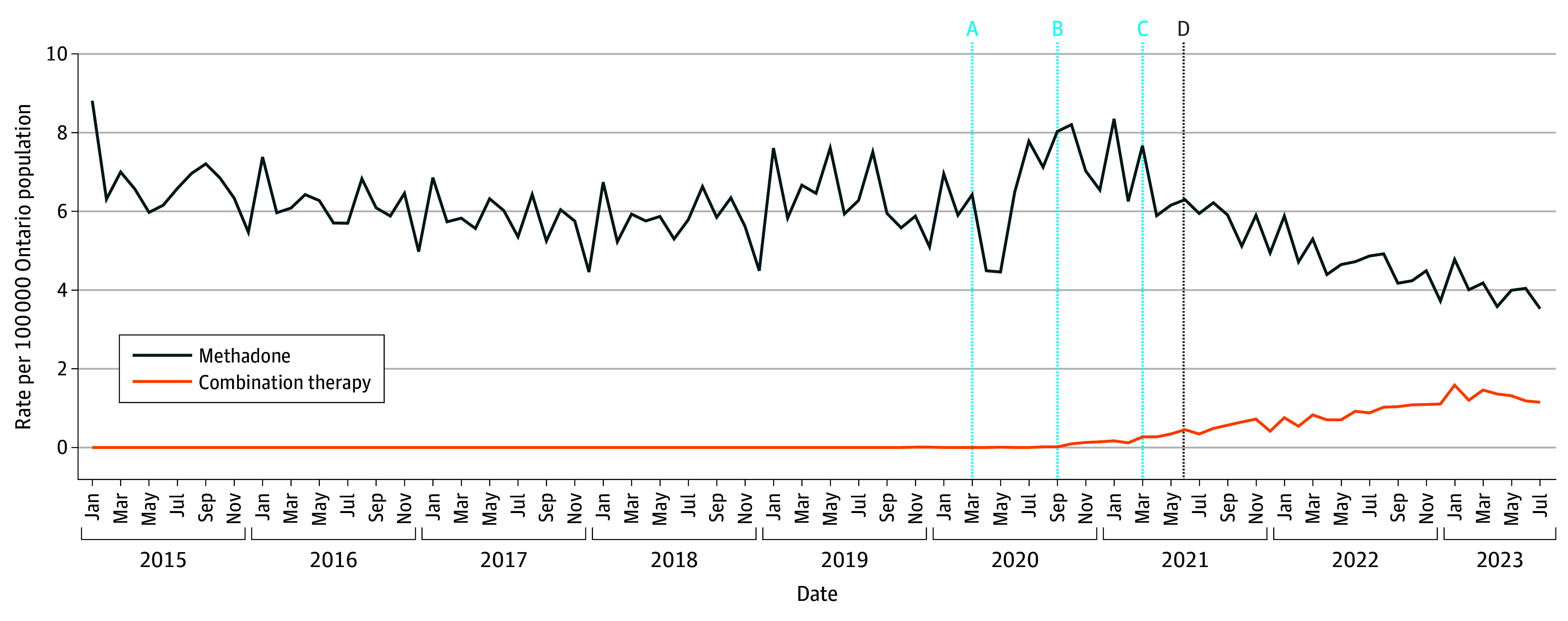
Monthly Methadone Initiation Rates, Stratified by Monotherapy and Combination Therapy in Ontario, Canada, January 2015 to July 2023 Vertical lines represent months of overlapping dates, with cyan lines representing intervention months included in interventional autoregressive integrated moving average models: A, declaration of emergency for COVID-19 in Ontario, Canada (March 2020); B, Mentoring, Education, and Clinical Tools for Addition–Partners in Health Integration (META-PHI) Annual Conference (September 2020); C, release of preliminary methadone prescribing recommendations by META-PHI and rescindment of College of Physician and Surgeons of Ontario guidelines; and D, official release of methadone prescribing recommendations by META-PHI.

From 2015 to 2019, methadone was initiated exclusively as monotherapy (Figure 1). A brief decline in monotherapy initiation rates was observed between March 2020 to May 2020, which coincided with the declaration of emergency for SARS-CoV-2 (pulse estimate, −1.53 per 100 000; 95% CI, −2.05 to −1.01 per 100 000) (Table 2). Although monotherapy initiation rates subsequently recovered, the presentation of new prescribing guidance at the META-PHI annual conference in September 2020 was associated with a sustained decline in methadone monotherapy initiation rates (ramp estimate, −0.27 per 100 000; 95% CI, −0.48 to −0.07 per 100 000; *P* = .01). Combination therapy initiation increased in tandem with declining monotherapy initiation rates. Fewer than 6 combination therapy episodes were observed before September 2020, but this number increased steadily in the following months, reaching 1.6 incident episodes per 100 000 population in January 2023. Despite this increase, monotherapy remained the predominant modality for methadone initiation (4.8 per 100 000 in January 2023).

**Table 2.** ARIMA Model Results

Outcome of interest	ARIMA model parameters, (p, d, q) × (P, D, Q)s	Interventions					
		SARS-CoV-2^a^		META-PHI conference^b^		Release of META-PHI guidance^c^	
		Estimate, % (95% CI)	*P* value	Estimate, % (95% CI)	*P* value	Estimate, % (95% CI)	*P* value
Methadone monotherapy initiation rates	(2,1,0) × (1,1,0)_12_^d^	−1.53 (−2.05 to −1.01)	<.001	−0.27 (−0.48 to −0.07)	.01	0.04 (−0.17 to 0.24)	.72
Monthly methadone dosing trends on index date							
<30 mg	(2,1,0)	NA	NA	−1.57 (−2.87 to −0.27)	.02	1.01 (−0.43 to 2.44)	.17
30 to <40 mg	(2,1,0)	NA	NA	1.59 (0.29 to 2.88)	.02	−1.40 (−2.83 to 0.02)	.05
40 to <50 mg	(3,1,0)	NA	NA	0.2 (−0.61 to 1.00)	.63	0.26 (−0.63 to 1.15)	.57
50 to <60 mg	(0,1,3)	NA	NA	−0.09 (−0.39 to 0.21)	.55	0.1 (−0.23 to 0.43)	.54
Monthly trends for maximum methadone dose dispensed between index date plus 1 d to index date plus 7 d							
<30 mg	(14,1,0)	NA	NA	−0.35 (−1.05 to 0.34)	.32	0.28 (−0.40 to 0.96)	.42
30 to <40 mg	(0,1,2)	NA	NA	0.79 (0.20 to 1.38)	.01	−0.88 (−1.52 to −0.24)	.01
40 to <50 mg	(0,1,1)	NA	NA	0.63 (0.13 to 1.13)	.01	−0.47 (−1.04 to 0.10)	.11
50 to <60 mg	(2,1,0)	NA	NA	0.04 (−0.30 to 0.38)	.83	0.05 (−0.33 to 0.42)	.80
≥60 mg	(0,1,1)	NA	NA	0.14 (0.04 to 0.24)	.005	−0.07 (−0.19 to 0.04)	.20
Monthly trends for maximum methadone dose dispensed between index-date plus 8 d to index date plus 13 d							
<30 mg	(3,1,0)	NA	NA	−0.10 (−0.63 to 0.42)	.71	0.00 (−0.58 to 0.58)	.99
30 to <40 mg	(0,1,1)	NA	NA	−0.27 (−0.62 to 0.09)	.14	0.2 (−0.20 to 0.60)	.34
40 to <50 mg	(0,1,2)	NA	NA	0.09 (−0.20 to 0.37)	.55	−0.09 (−0.41 to 0.22)	.57
50 to <60 mg	(0,1,1)	NA	NA	−0.37 (−0.62 to −0.12)	.003	0.34 (0.05 to 0.63)	.02
≥60 mg	(0,1,1)	NA	NA	0.71 (0.25 to 1.17)	.002	−0.58 (−1.09 to −0.07)	.03

Abbreviations: ARIMA, autoregressive intervention moving average; META-PHI, Mentoring, Education, and Clinical Tools for Addition–Partners in Health Integration; NA, not applicable.

^a^ Refers to declaration of the SARS-CoV-2 public health emergency in March 2020.

^b^ Refers to presentation of new methadone prescribing recommendations at META-PHI Annual Conference in September 2020.

^c^ Refers to release of draft guidance document by META-PHI, outlining new methadone prescribing guidance in March 2021.

^d^ The 12 refers to seasonal differencing.

**Figure 2.**
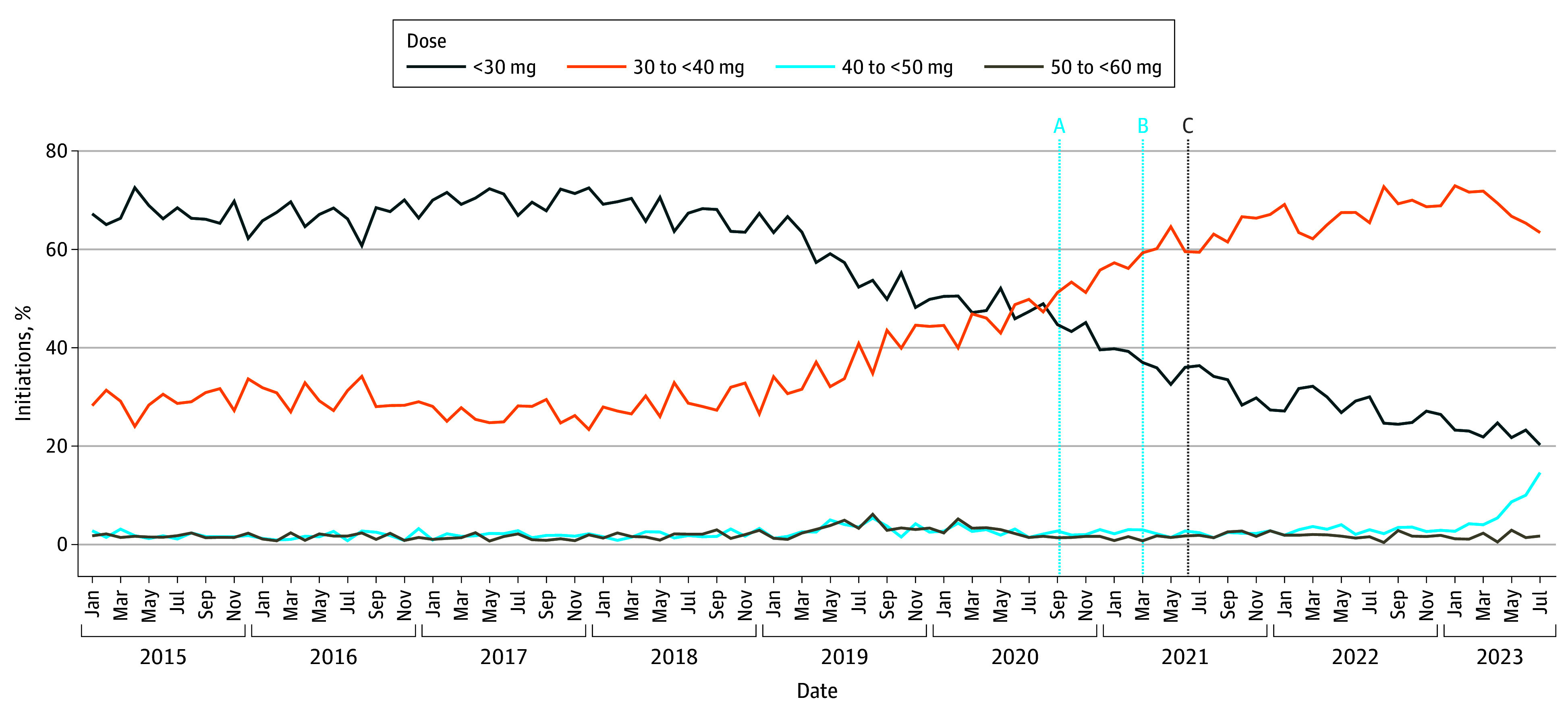
Methadone Dose Dispensed on the First Day of Treatment Among Incident Methadone Recipients in Ontario, Canada, January 2015 to July 2023 Vertical lines represent months of overlapping dates, with cyan lines representing intervention months included in interventional autoregressive integrated moving average models: A, Mentoring, Education, and Clinical Tools for Addition–Partners in Health Integration (META-PHI) Annual Conference (September 2020); B, release of preliminary methadone prescribing recommendations by META-PHI and rescindment of College of Physician and Surgeons of Ontario guidelines; and C, official release of methadone prescribing recommendations by META-PHI.

A shift toward attainment of higher methadone doses within the first 2 weeks of treatment was observed following 2018 (Figure 2 and eFigures 2 and 3 in Supplement 1). Between 2015 and 2017, 60.8% (466 episodes) to 72.6% (526 episodes) of initiations were dispensed less than 30 mg of methadone at index. In 2018, the proportion of episodes initiated at doses less than 30 mg declined, which coincided with increased initiation at doses between 30 to less than 40 mg. Following the 2020 META-PHI annual conference the proportion of methadone episodes dispensed less than 30 mg on index declined by an additional 1.57% per month (95% CI, −2.87% to −0.27%; *P* = .02) (Table 2). Concurrently, the proportion of episodes dispensed doses between 30 to less than 40 mg on index significantly increased (ramp estimate, 1.59%; 95% CI, 0.29% to 2.88%). By the end of the study period, 63.4% of methadone episodes (369 episodes) were dispensed doses between 30 to less than 40 mg on initiation, compared with 28.2% (274 episodes) in January 2015. Of note, among episodes initiated at doses between 30 to less than 40 mg over the study period, 98.0% (25 493 episodes) were dispensed 30 mg at index (eTable 4 in Supplement 1). Finally, the proportion of episodes initiated at doses between 40 and less than 50 mg increased 4-fold in 2023, from 2.7% (21 episodes) in January to 14.6% (85 episodes) in July.

In the first week of treatment, we observed an increase in the proportion of methadone episodes that were dispensed doses between 40 to less than 50 mg (eFigure 2 in Supplement 1). Between 2015 and 2017, only 8.6% (44 episodes) to 18.5% (142 episodes) of episodes reached doses between 40 and less than 50 mg in the first week. Following 2018, this trend began to increase, reaching 17.3% (165 episodes) in September 2020, and was further accelerated by an additional 0.63% per month (95% CI, 0.13% to 1.13%; *P* = .01), thereafter reaching 27.3% of all episodes (159 episodes) by July 2023. Similarly, the proportion of methadone episodes dispensed doses greater than or equal to 60 mg in week 2 increased after 2018 and the trend further accelerated following the 2020 META-PHI conference (ramp estimate, 0.71%; 95% CI, 0.25% to 1.17%; *P* = .002). In July 2023, 18.9% of methadone initiations (110 episodes) achieved doses greater than or equal to 60 mg by the second week of treatment, compared with 10.9% (106 episodes) in January 2015.

Medication use patterns following initiation were reported overall and stratified by medications (Table 3) and methadone dose dispensed on index (eTables 5 and 6 in Supplement 1). Overall, 0.9% of monotherapy initiations (667 episodes) were dispensed SROM within 13 days of initiation. A significant decrease in the provision of rapid dose titration and treatment retention was noted. Specifically, although 42.8% of monotherapy episodes (20 434 episodes) initiated before September 2020 received their first dose increase within 6 days, this significantly decreased to 37.5% (8500 episodes) thereafter. Moreover, the prevalence of episodes not provided any dose titration in the first 2 weeks significantly increased from 38.5% (18 390 episodes) to 45.7% (10 416 episodes) over the same period. Dose titration was even more limited among combination therapy episodes, with a significantly lower prevalence of titration within 6 days (896 episodes [29.2%] vs 8500 episodes [37.5%]), and increased proportion not provided any dose increases in the first 2 weeks (1580 episodes [51.5%] vs 10 416 episodes [45.7%]) compared with monotherapy episodes initiated in September 2020 or later. Among those provided a dose increase, the absolute amount methadone was titrated within the first 2 weeks was generally stable over time.

**Table 3.** Medication Use Characteristics During the First 2 Weeks of Treatment, Reported Overall and Stratified by Medication Dispensed on Index Date

Variable	Episodes, No. (%)			
	Overall	Methadone before September 2020	Methadone September 2020 and later	Combination therapy (methadone and SROM)
Continuous use periods, No.	73 633	47 779	22 785	3069
Unique individuals, No.	35 309	27 101	14 097	2057
Provision of combination therapy within 13 d following index date	667 (0.9)	16 (<0.1)^a^	651 (2.9)	NA
Dose dispensed on index date				
<30 mg	38 446 (52.2)	30 302 (63.4)^a^	7964 (35.0)	180 (5.9)^b^
30 to <40 mg	31 870 (43.3)	15 447 (32.3)^a^	13 573 (59.6)	2850 (92.9)^b^
40 to <50 mg	1911 (2.6)	1050 (2.2)	828 (3.6)	33 (1.1)^b^
50 to <60 mg	1406 (1.9)	980 (2.1)	420 (1.8)	6 (0.2)^b^
Date of first dose increase				
Index date plus 1 d to index date plus 5 d	29 830 (40.5)	20 434 (42.8)^a^	8500 (37.5)	896 (29.2)^b^
Index date plus 6 d to index date plus 13 d	13 417 (18.2)	8955 (18.7)	3869 (17.0)	593 (19.3)
No dose increase	30 386 (41.3)	18 390 (38.5)^a^	10 416 (45.7)	1580 (51.5)^b^
Dose difference between maximum methadone dose dispensed in week 2 and on initiation				
No dose dispensed in interval 3	19 494 (26.5)	11 175 (23.4)^a^	7221 (31.7)	1098 (35.8)
No change	12 076 (16.4)	7729 (16.2)	3758 (16.5)	589 (19.2)
Dose decrease	1758 (2.4)	1227 (2.6)	522 (2.3)	9 (0.3)^b^
>0 to <15 mg	12 362 (16.8)	9458 (19.8)^a^	2779 (12.2)	125 (4.1)^b^
15 to <30 mg	16 261 (22.1)	11 139 (23.3)	4465 (19.6)	657 (21.4)
30 to <44 mg	8923 (12.1)	5681 (11.9)	2821 (12.4)	421 (13.7)
≥45 mg	2759 (3.7)	1370 (2.9)^a^	1219 (5.4)	170 (5.5)
Maximum dose dispensed during treatment days 2-7				
<30 mg	20 356 (27.6)	16 362 (34.2)^a^	3906 (17.1)	88 (2.9)^b^
30 to <40 mg	27 207 (36.9)	16 643 (34.8)	8970 (39.4)	1594 (51.9)^b^
40 to <50 mg	12 977 (17.6)	7209 (15.1)^a^	4972 (21.8)	796 (25.9)
50 to <60 mg	1802 (2.4)	1143 (2.4)	636 (2.8)	23 (0.7)^b^
≥60 mg	1779 (2.4)	916 (1.9)	760 (3.3)	103 (3.4)
No methadone dispensed in interval 2	9512 (12.9)	5506 (11.5)^a^	3541 (15.5)	465 (15.2)
Maximum dose dispensed during treatment days 8-14				
<30 mg	10 017 (13.6)	7903 (16.5)^a^	2083 (9.1)	31 (1.0)^b^
30 to <40 mg	15 428 (21.0)	10 525 (22.0)	4276 (18.8)	627 (20.4)
40 to <50 mg	13 641 (18.5)	9063 (19.0)	3940 (17.3)	638 (20.8)
50 to <60 mg	6745 (9.2)	4968 (10.4)^a^	1688 (7.4)	89 (2.9)^b^
≥60 mg	8308 (11.3)	4145 (8.7)^a^	3577 (15.7)	586 (19.1)
No methadone dispensed in interval 3	19 494 (26.5)	11 175 (23.4)^a^	7221 (31.7)	1098 (35.8)

Abbreviations: NA, not applicable; SROM, slow-release oral morphine.

^a^ Indicates observation of a meaningful difference based on a standardized difference greater than 0.10 between those who initiated methadone prior to September 2020 vs in September 2020 or later.

^b^ Indicates observation of a meaningful difference based on a standardized difference greater than 0.10 between those who initiated methadone monotherapy in September 2020 or later vs combination therapy.

## Discussion

In this cross-sectional, population-based study examining methadone prescribing trends, before 2018, methadone was generally initiated as monotherapy, and at doses less than 30 mg. In 2018, a shift toward initiation of methadone at 30 mg was observed, which led to attainment of higher doses within the first 2 weeks of treatment. The announcement of new methadone prescribing guidance at the 2020 META-PHI conference further accelerated the shift toward higher dosing and uptake of methadone initiation with SROM. Although a shift in methadone initiation patterns was noted, subsequent provision of dose titration declined, representing a potential missed opportunity for quicker attainment of therapeutic doses.

Our study noted adoption of new methadone initiation regimens, including higher starting doses and induction with SROM. Traditionally used for chronic pain, off-label use of SROM as OAT is a novel practice recently adopted in Canada.^20^ Although further research is needed on the safety of concurrent full-opioid agonist use, emerging evidence indicates improved treatment retention among those provided OAT with another full opioid agonist (eg, and SROM) compared with monotherapy.^21^ Despite an increase in methadone dose dispensed at initiation, ongoing provision of dose titration within the first 2 weeks was limited. Specifically, among methadone monotherapy episodes initiated in September 2020 or after, only 37.5% received a dose increase within the first 6 days of starting treatment, and 45.7% received no dose increase in the first 2 weeks. For patients tolerant to fentanyl and at low risk of toxicity, the META-PHI recommends dose increases of 10 to 15 mg every 3 to 5 days, for maximum titration of 45 mg within the first 14 days.^6^ The low prevalence of dose titrations and continued provision of doses at the lower end of the recommended titration schedule suggest a missed opportunity for optimal methadone use. People who use fentanyl often require methadone doses greater than 100 mg per day to mitigate symptoms of cravings and withdrawal.^6^ Prolonged periods taking subtherapeutic doses may result in treatment dissatisfaction, leading to abrupt discontinuation and potential return to the unregulated drug supply. The lack of rapid dose titration may stem from various factors, including prescriber discomfort due to limited evidence supporting this practice alongside high initiation doses and logistical barriers (eg, requirements for in-person physician visits before dose titration). Although outpatient methadone titration may be logistically challenging, emerging evidence from a case series conducted in San Francisco, California, supports the feasibility of more aggressive dose titration protocols for patients exposed to fentanyl in outpatient treatment settings, with most participants achieving a dose of 80 mg by treatment day 7 and no evidence of oversedation or overdose.^22^

A 2024 observational study conducted in British Columbia, Canada reported increasing initiation of OAT at higher doses, more-rapid dose titration, and maintenance at higher doses over time.^23^ However, some notable differences in methadone prescribing practices between British Columbia and Ontario were observed. In British Columbia, one-third of incident methadone episodes in 2021 were dispensed more than 30 mg on the first day of treatment, and nearly 40% received their first dose increase in under 5 days.^23^ Interprovincial differences in methadone prescribing patterns across Canada may reflect variations in the unregulated drug supply,^1,24^ the burden of opioid-related harms,^1^ and OAT prescribing guidance.^6,25^ British Columbia, where fentanyl first entered the national unregulated drug supply, reports the highest population-adjusted rates of opioid-related harms across Canada. Elevated potency of the unregulated drug supply in British Columbia likely led to early adoption of higher starting doses and rapid dose titration by prescribers. Although methadone initiation at 30 mg remained the dominant dose throughout our study period, in 2023, the proportion of episodes initiated at 40 mg increased 4-fold, likely driven by patient-reported opioid tolerance and the influence of methadone prescribing guidance from British Columbia and the US.^25,26^

### Limitations

Study limitations that warrant discussion include our inability to capture medication dispensed out-of-province or within a correctional facility, which may result in misclassification of incident methadone episodes. The severity of Ontario’s opioid toxicity epidemic has also escalated over our study period, precipitating the implementation of different policies and clinical interventions. Therefore, causal inferences cannot be established on the basis of our ARIMA models owing to the possibility of additional events occurring in close proximity to included intervention dates. Furthermore, our study was conducted in a single Canadian province. However, given similarities in the fentanyl-dominated unregulated drug supply and elevated patient-reported opioid tolerance across Canada and the US, our findings may be reflective of evolving patterns in other jurisdictions.

## Conclusions

The increased potency of the unregulated drug supply and resultant elevated opioid tolerance among people with OUD have precipitated initiation of methadone at higher doses and in combination with SROM. Although attainment of higher methadone doses by the second week of treatment was noted, this was largely driven by provision of higher starting doses, whereas uptake of rapid dose titration remained limited. This suggests a missed opportunity for faster attainment of therapeutic doses for people with a high opioid tolerance. Reasons for the limited provision of dose titration in the first 2 weeks of treatment should be further investigated to support the adoption of rapid dose titration protocols and improve treatment retention. Furthermore, future research should investigate the safety and effectiveness of novel methadone prescribing regimens to support development of evidence-based prescribing practices.

## Notes

Supplement 1. **eTable 1.** Description of All Linked Administrative Databases Used in the Study **eTable 2.** Drug Identification Numbers **eTable 3.** Covariate Definitions **eTable 4.** Maximum Methadone Dose Dispensed Among Incident Recipient of Methadone, Stratified by Time on Treatment **eTable 5.** Medication Use Characteristics During the First Two Weeks of Treatment, Reported Overall and Stratified by Methadone Dose Dispensed on Index-Date **eTable 6.** Baseline Characteristics, Reported Overall and Stratified by Methadone Dose Dispensed on Index-Date **eFigure 1.** Cohort Exclusion Flow Diagram **eFigure 2.** Maximum Methadone Dose Dispensed Between the Second to Seventh Day of Treatment Among Incident Methadone Recipients in Ontario, Canada, Between January 2015 to July 2023 **eFigure 3.** Maximum Methadone Dose Dispensed Between the Eighth to 14th Day of Treatment Among Incident Methadone Recipients in Ontario, Canada, Between January 2015 to July 2023

Supplement 2.
Data Sharing Statement
