# Supplementary material for: Initiation and Dose of Methadone Monotherapy vs Combination Therapy, 2015 to 2023
Source: JAMA Netw Open. 2025 Aug 15;8(8):e2527290. doi: 10.1001/jamanetworkopen.2025.27290 (PMC12357187; doi:10.1001/jamanetworkopen.2025.27290)
Supplement: Supplement 1. — eTable 1. Description of All Linked Administrative Databases Used in the Study eTable 2. Drug Identification Numbers eTable 3. Covariate Definitions eTable 4. Maximum Methadone Dose Dispensed Among Incident Recipient of Methadone, Stratified by Time on Treatment eTable 5. Medication Use Characteristics During the First Two Weeks of Treatment, Reported Overall and Stratified by Methadone Dose Dispensed on Index-Date eTable 6. Baseline Characteristics, Reported Overall and Stratified by Methadone Dose Dispensed on Index-Date eFigure 1. Cohort Exclusion Flow Diagram eFigure 2. Maximum Methadone Dose Dispensed Between the Second to Seventh Day of Treatment Among Incident Methadone Recipients in Ontario, Canada, Between January 2015 to July 2023 eFigure 3. Maximum Methadone Dose Dispensed Between the Eighth to 14th Day of Treatment Among Incident Methadone Recipients in Ontario, Canada, Between January 2015 to July 2023 [file jamanetwopen-e2527290-s001.pdf]

Supplemental Online Content

Garg R, Luo J, Bozinoff N, et al. Initiation and dose of methadone monotherapy vs combination therapy, 2015 to 2023. *JAMA Netw Open*. 2025;8(8):e2527290. doi:10.1001/jamanetworkopen.2025.27290

eTable 1. Description of All Linked Administrative Databases Used in the Study
eTable 2. Drug Identification Numbers
eTable 3. Covariate Definitions
eTable 4. Maximum Methadone Dose Dispensed Among Incident Recipient of Methadone, Stratified by Time on Treatment
eTable 5. Medication Use Characteristics During the First Two Weeks of Treatment, Reported Overall and Stratified by Methadone Dose Dispensed on Index-Date
eTable 6. Baseline Characteristics, Reported Overall and Stratified by Methadone Dose Dispensed on Index-Date
eFigure 1. Cohort Exclusion Flow Diagram
eFigure 2. Maximum Methadone Dose Dispensed Between the Second to Seventh Day of Treatment Among Incident Methadone Recipients in Ontario, Canada, Between January 2015 to July 2023
eFigure 3. Maximum Methadone Dose Dispensed Between the Eighth to 14th Day of Treatment Among Incident Methadone Recipients in Ontario, Canada, Between January 2015 to July 2023

This supplemental material has been provided by the authors to give readers additional information about their work.

© 2025 Garg R et al. *JAMA Network Open*.

eTable 1. Description of All Linked Administrative Databases Used in the Study

**Database**	**Description**
Narcotics Monitoring System (NMS)	Captures all prescriptions for controlled substances dispensed from community pharmacies in Ontario, regardless of insurance status. This database was used to identify opioid agonist therapy dispensations.
Registered Persons Database (RPDB)	Captures demographic and vital status characteristics for everyone eligible for the publicly-funded Ontario Health Insurance Plan (OHIP).
Canadian Institute for Health Information (CIHI) Discharge Abstract Database (DAD)	Captures details on diagnoses and procedures for all inpatient hospital stays in Ontario.
CIHI National Ambulatory Care Reporting System (NACRS)	Captures details on diagnoses and procedures for all emergency department visits in Ontario.
CIHI Ontario Mental Health Reporting System (OMHRS)	Captures details on diagnoses and procedures for all inpatient stays in mental health hospitals in Ontario.
Ontario Health Insurance Plan (OHIP) Database	Captures outpatient care using billing information for all services covered by OHIP in Ontario.
ICES Physician Database (IPDB)	Captures information on physcians eligible to receive payment from the OHIP, such as demographics, training, and practice location.
Ontario Cancer Registry (OCR)	A provincial registry of all incident cancer diagnoses in Ontario.
Cancer Activity Level Reporting (ALR)	Captures details regarding inpatient and outpatient provision of radiation and systhemic therapy services for cancer treatment.
New Drug Funding Program (NDFP)	Captures prescription for expensive drugs (e.g., outpatient cancer medications) reimbursed by the the provincial drug program.

© 2025 Garg R et al. *JAMA Network Open*.

eTable 2. Drug Identification Numbers

Drug Identification Number	Drug Chemical Name	Drug Brand Name
2474921	BUPRENORPHINE HCL	Probuphine
2483084	BUPRENORPHINE	Sublocade
2483092	BUPRENORPHINE	Sublocade
9858127	BUPRENORPHINE	Sublocade
9858128	BUPRENORPHINE	Sublocade
2295695	BUPRENORPHINE HCL & NALOXONE HCL	Suboxone
2295709	BUPRENORPHINE HCL & NALOXONE HCL	Suboxone
2408090	BUPRENORPHINE HCL & NALOXONE HCL	Mylan-Buprenorphine/Naloxone
2408104	BUPRENORPHINE HCL & NALOXONE HCL	Mylan-Buprenorphine/Naloxone
2424851	BUPRENORPHINE HCL & NALOXONE HCL	Teva-Buprenorphine/Naloxone
2424878	BUPRENORPHINE HCL & NALOXONE HCL	Teva-Buprenorphine/Naloxone
9857539	BUPRENORPHINE	Subutex
2453908	BUPRENORPHINE HCL & NALOXONE HCL	Act Buprenorphine/Naloxone
2453916	BUPRENORPHINE HCL & NALOXONE HCL	Act Buprenorphine/Naloxone
2468085	BUPRENORPHINE HCL & NALOXONE HCL	Suboxone
2468093	BUPRENORPHINE HCL & NALOXONE HCL	Suboxone
2502356	BUPRENORPHINE HCL & NALOXONE HCL	Suboxone
2502313	BUPRENORPHINE HCL & NALOXONE HCL	Suboxone
2502321	BUPRENORPHINE HCL & NALOXONE HCL	Suboxone
2502348	BUPRENORPHINE HCL & NALOXONE HCL	Suboxone
02319446	HYDROMORPHONE HCL	Teva-Hydromorphone
00885428	HYDROMORPHONE HCL	PMS-HYDROmorphone
02192144	HYDROMORPHONE HCL	Hydromorphone
02245705	HYDROMORPHONE HCL	Hydromorph.IR - 8mg
00786543	HYDROMORPHONE HCL	Dilaudid
02364158	HYDROMORPHONE HCL	Apo-Hydromorphone
02319438	HYDROMORPHONE HCL	Teva-Hydromorphone
00885401	HYDROMORPHONE HCL	PMS-HYDROmorphone
02249936	HYDROMORPHONE HCL	Hydromorphone
02245704	HYDROMORPHONE HCL	Hydromorph.IR - 4mg
00125121	HYDROMORPHONE	Dilaudid

© 2025 Garg R et al. *JAMA Network Open*.

02364131	HYDROMORPHONE HCL	Apo-Hydromorphone
2244290	METHADONE HCL	Metadol-D
2394596	METHADONE HCL	Methadose
2394618	METHADONE HCL	Methadose
9850619	METHADONE	Methadone
9857499	METHADONE MIXTURE	Methadone Compounding for MMT
2481979	METHADONE HCL	Methadone HCl Oral Concentrate USP
2495872	METHADONE HCL	Odan-Methadone
2495880	METHADONE HCL	Odan-Methadone
2495783	METHADONE HCL	Jamp Methadone Oral Concentrate
2184435	MORPHINE SULFATE	Kadian
2184443	MORPHINE SULFATE	Kadian
2184451	MORPHINE SULFATE	Kadian
2242163	MORPHINE SULFATE	Kadian
2019930	MORPHINE SULFATE	M-Eslon
2019949	MORPHINE SULFATE	M-Eslon
2019957	MORPHINE SULFATE	M-Eslon
2019965	MORPHINE SULFATE	M-Eslon
2177749	MORPHINE SULFATE	M-Eslon
2177757	MORPHINE SULFATE	M-Eslon

© 2025 Garg R et al. *JAMA Network Open*.

eTable 3. Covariate Definitions

**Variable**	**Database**	**Definition**
**Patient-related covariates**		
Age	RPDB	Age at index using birth date in RPDB
Sex	RPDB	Sex as recorded in RPDB
Rurality of residence	RPDB	Rurality of residence using person's postal code
Neighbourhood income quintile	RPDB	Neighbourhood income quintile using person's postal code
Prescribed medications 180 days before index	NMS	A dispense record for specified medications in the 180 days prior to index date
Occurrence of opioid toxicity in 365-days prior to index date	DAD, NACRS	ED visit or inpatient hospitalization with diagnosis of opioid poisoning using codes below<br>**ICD-10 Codes:** T40.0, T40.1, T40.2, T40.3, T40.4, T40.6
**Prescriber-related covariates**		
Prescriber type	NMS, IPDB	Prescriber identified on the methadone dispense record on the index date
OAT prescriber volume	NMS, IPDB	Defined based on the distribution of unique OAT clients per prescriber identified in our study cohort

© 2025 Garg R et al. *JAMA Network Open*.

eTable 4. Maximum Methadone Dose Dispensed Among Incident Recipient of Methadone, Stratified by Time on Treatment

**Year**	2015	2016	2017	2018	2019	2020	2021	2022	2023
**Incident methadone continuous use periods**	8879	8268	7891	7987	8904	9443	9517	8123	4621
Interval 1 (i.e., index-date)									
1 to <15mg	659 (7.4%)	643 (7.8%)	603 (7.6%)	493 (6.2%)	518 (5.8%)	360 (3.8%)	356 (3.7%)	279 (3.4%)	127 (2.7%)
15mg	724 (8.2%)	609 (7.4%)	626 (7.9%)	499 (6.2%)	441 (5.0%)	377 (4.0%)	280 (2.9%)	191 (2.4%)	102 (2.2%)
>15 to <20mg	102 (1.1%)	140 (1.7%)	183 (2.3%)	208 (2.6%)	206 (2.3%)	236 (2.5%)	105 (1.1%)	69 (0.8%)	30 (0.6%)
20mg	3653 (41.1%)	3486 (42.2%)	3548 (45.0%)	3504 (43.9%)	3031 (34.0%)	2510 (26.6%)	1671 (17.6%)	1003 (12.3%)	482 (10.4%)
>20 to <25mg	63 (0.7%)	57 (0.7%)	47 (0.6%)	57 (0.7%)	89 (1.0%)	124 (1.3%)	127 (1.3%)	115 (1.4%)	62 (1.3%)
25mg	733 (8.3%)	558 (6.7%)	514 (6.5%)	574 (7.2%)	692 (7.8%)	676 (7.2%)	560 (5.9%)	421 (5.2%)	162 (3.5%)
>25 to <30mg	22 (0.2%)	31 (0.4%)	37 (0.5%)	41 (0.5%)	65 (0.7%)	116 (1.2%)	182 (1.9%)	187 (2.3%)	80 (1.7%)
30mg	2524 (28.4%)	2406 (29.1%)	2002 (25.4%)	2237 (28.0%)	3148 (35.4%)	4506 (47.7%)	5755 (60.5%)	5398 (66.5%)	3096 (67.0%)
>30 to <35mg	9 (0.1%)	18 (0.2%)	20 (0.3%)	15 (0.2%)	27 (0.3%)	13 (0.1%)	15 (0.2%)	22 (0.3%)	34 (0.7%)
35mg	53 (0.6%)	37 (0.4%)	46 (0.6%)	37 (0.5%)	81 (0.9%)	62 (0.7%)	63 (0.7%)	38 (0.5%)	35 (0.8%)
>35 to <40mg	18 (0.2%)	14 (0.2%)	14 (0.2%)	16 (0.2%)	32 (0.4%)	12 (0.1%)	21 (0.2%)	24 (0.3%)	22 (0.5%)
40mg	87 (1.0%)	64 (0.8%)	77 (1.0%)	82 (1.0%)	124 (1.4%)	125 (1.3%)	124 (1.3%)	147 (1.8%)	246 (5.3%)
>40 to <45mg	9 (0.1%)	13 (0.2%)	11 (0.1%)	8 (0.1%)	24 (0.3%)	16 (0.2%)	10 (0.1%)	12 (0.1%)	3 (0.1%)
45mg	58 (0.7%)	52 (0.6%)	46 (0.6%)	53 (0.7%)	121 (1.4%)	90 (1.0%)	77 (0.8%)	66 (0.8%)	58 (1.3%)
>45 to <50mg	13 (0.1%)	13 (0.2%)	13 (0.2%)	12 (0.2%)	17 (0.2%)	8 (0.1%)	11 (0.1%)	12 (0.1%)	9 (0.2%)
50mg	85 (1.0%)	69 (0.8%)	60 (0.8%)	76 (1.0%)	173 (1.9%)	109 (1.2%)	96 (1.0%)	88 (1.1%)	38 (0.8%)
>50 to <55mg	10 (0.1%)	8 (0.1%)	3 (0.0%)	11 (0.1%)	16 (0.2%)	12 (0.1%)	10 (0.1%)	4 (0.0%)	5 (0.1%)
55mg	49 (0.6%)	43 (0.5%)	37 (0.5%)	51 (0.6%)	77 (0.9%)	78 (0.8%)	46 (0.5%)	39 (0.5%)	29 (0.6%)
>55 to 59mg	8 (0.1%)	7 (0.1%)	4 (0.1%)	13 (0.2%)	22 (0.2%)	13 (0.1%)	8 (0.1%)	8 (0.1%)	1 (0.0%)
Interval 2 (i.e., index-date+1 to index-date+6)									
1 to <15mg	281 (3.2%)	283 (3.4%)	255 (3.2%)	219 (2.7%)	231 (2.6%)	170 (1.8%)	162 (1.7%)	131 (1.6%)	69 (1.5%)
15mg	289 (3.3%)	265 (3.2%)	288 (3.6%)	237 (3.0%)	207 (2.3%)	160 (1.7%)	132 (1.4%)	104 (1.3%)	60 (1.3%)
>15 to <20mg	58 (0.7%)	67 (0.8%)	59 (0.7%)	45 (0.6%)	51 (0.6%)	38 (0.4%)	19 (0.2%)	20 (0.2%)	7 (0.2%)
20mg	1537 (17.3%)	1577 (19.1%)	1751 (22.2%)	1721 (21.5%)	1494 (16.8%)	1214 (12.9%)	827 (8.7%)	507 (6.2%)	238 (5.2%)
>20 to <25mg	75 (0.8%)	98 (1.2%)	73 (0.9%)	84 (1.1%)	66 (0.7%)	89 (0.9%)	77 (0.8%)	69 (0.8%)	30 (0.6%)
25mg	883 (9.9%)	719 (8.7%)	616 (7.8%)	568 (7.1%)	535 (6.0%)	480 (5.1%)	334 (3.5%)	223 (2.7%)	93 (2.0%)

© 2025 Garg R et al. *JAMA Network Open*.

>25 to <30mg	66 (0.7%)	65 (0.8%)	51 (0.6%)	54 (0.7%)	71 (0.8%)	60 (0.6%)	42 (0.4%)	31 (0.4%)	31 (0.7%)
30mg	2415 (27.2%)	2287 (27.7%)	2187 (27.7%)	2273 (28.5%)	2525 (28.4%)	3099 (32.8%)	3446 (36.2%)	3070 (37.8%)	1690 (36.6%)
>30 to <35mg	39 (0.4%)	41 (0.5%)	28 (0.4%)	20 (0.3%)	40 (0.4%)	36 (0.4%)	38 (0.4%)	34 (0.4%)	19 (0.4%)
35mg	582 (6.6%)	493 (6.0%)	445 (5.6%)	444 (5.6%)	466 (5.2%)	437 (4.6%)	367 (3.9%)	238 (2.9%)	93 (2.0%)
>35 to <40mg	52 (0.6%)	22 (0.3%)	15 (0.2%)	23 (0.3%)	30 (0.3%)	42 (0.4%)	78 (0.8%)	61 (0.8%)	32 (0.7%)
40mg	787 (8.9%)	668 (8.1%)	560 (7.1%)	577 (7.2%)	614 (6.9%)	624 (6.6%)	533 (5.6%)	412 (5.1%)	328 (7.1%)
>40 to <45mg	11 (0.1%)	12 (0.1%)	9 (0.1%)	11 (0.1%)	18 (0.2%)	17 (0.2%)	24 (0.3%)	22 (0.3%)	13 (0.3%)
45mg	638 (7.2%)	572 (6.9%)	440 (5.6%)	480 (6.0%)	743 (8.3%)	1052 (11.1%)	1495 (15.7%)	1413 (17.4%)	815 (17.6%)
>45 to <50mg	11 (0.1%)	9 (0.1%)	15 (0.2%)	5 (0.1%)	11 (0.1%)	9 (0.1%)	9 (0.1%)	10 (0.1%)	10 (0.2%)
50mg	139 (1.6%)	114 (1.4%)	104 (1.3%)	108 (1.4%)	158 (1.8%)	144 (1.5%)	111 (1.2%)	101 (1.2%)	52 (1.1%)
>50 to <55mg	6 (0.1%)	6 (0.1%)	3 (0.0%)	9 (0.1%)	14 (0.2%)	6 (0.1%)	14 (0.1%)	6 (0.1%)	6 (0.1%)
55mg	62 (0.7%)	51 (0.6%)	40 (0.5%)	65 (0.8%)	67 (0.8%)	95 (1.0%)	89 (0.9%)	91 (1.1%)	91 (2.0%)
>55 to 59mg	7 (0.1%)	2 (0.0%)	1 (0.0%)	6 (0.1%)	6 (0.1%)	9 (0.1%)	5 (0.1%)	9 (0.1%)	5 (0.1%)
60mg	59 (0.7%)	66 (0.8%)	54 (0.7%)	62 (0.8%)	92 (1.0%)	110 (1.2%)	186 (2.0%)	194 (2.4%)	120 (2.6%)
>60 to 100mg	12 (0.1%)	16 (0.2%)	15 (0.2%)	10 (0.1%)	25 (0.3%)	27 (0.3%)	32 (0.3%)	22 (0.3%)	29 (0.6%)
>100mg	140 (1.6%)	94 (1.1%)	68 (0.9%)	67 (0.8%)	40 (0.4%)	44 (0.5%)	61 (0.6%)	81 (1.0%)	53 (1.1%)
Interval 3 (i.e., index-date+7 to index-date+13)									
1 to <15mg	169 (1.9%)	149 (1.8%)	143 (1.8%)	129 (1.6%)	158 (1.8%)	117 (1.2%)	105 (1.1%)	89 (1.1%)	46 (1.0%)
15mg	157 (1.8%)	129 (1.6%)	150 (1.9%)	125 (1.6%)	106 (1.2%)	90 (1.0%)	74 (0.8%)	75 (0.9%)	50 (1.1%)
>15 to <20mg	71 (0.8%)	65 (0.8%)	52 (0.7%)	51 (0.6%)	45 (0.5%)	33 (0.3%)	27 (0.3%)	28 (0.3%)	15 (0.3%)
20mg	608 (6.8%)	631 (7.6%)	668 (8.5%)	694 (8.7%)	581 (6.5%)	448 (4.7%)	358 (3.8%)	232 (2.9%)	110 (2.4%)
>20 to <25mg	65 (0.7%)	46 (0.6%)	41 (0.5%)	52 (0.7%)	43 (0.5%)	37 (0.4%)	22 (0.2%)	26 (0.3%)	8 (0.2%)
25mg	474 (5.3%)	402 (4.9%)	347 (4.4%)	301 (3.8%)	283 (3.2%)	276 (2.9%)	207 (2.2%)	167 (2.1%)	50 (1.1%)
>25 to <30mg	53 (0.6%)	59 (0.7%)	51 (0.6%)	58 (0.7%)	56 (0.6%)	42 (0.4%)	26 (0.3%)	26 (0.3%)	21 (0.5%)
30mg	1323 (14.9%)	1278 (15.5%)	1235 (15.7%)	1272 (15.9%)	1324 (14.9%)	1575 (16.7%)	1531 (16.1%)	1273 (15.7%)	713 (15.4%)
>30 to <35mg	52 (0.6%)	62 (0.7%)	43 (0.5%)	24 (0.3%)	39 (0.4%)	32 (0.3%)	25 (0.3%)	22 (0.3%)	9 (0.2%)
35mg	597 (6.7%)	506 (6.1%)	464 (5.9%)	437 (5.5%)	370 (4.2%)	348 (3.7%)	259 (2.7%)	180 (2.2%)	87 (1.9%)
>35 to <40mg	55 (0.6%)	56 (0.7%)	38 (0.5%)	46 (0.6%)	40 (0.4%)	41 (0.4%)	28 (0.3%)	25 (0.3%)	19 (0.4%)
40mg	1123 (12.6%)	1002 (12.1%)	936 (11.9%)	877 (11.0%)	830 (9.3%)	763 (8.1%)	581 (6.1%)	408 (5.0%)	223 (4.8%)
>40 to <45mg	39 (0.4%)	30 (0.4%)	23 (0.3%)	29 (0.4%)	33 (0.4%)	36 (0.4%)	23 (0.2%)	19 (0.2%)	15 (0.3%)
45mg	688 (7.7%)	597 (7.2%)	512 (6.5%)	526 (6.6%)	665 (7.5%)	833 (8.8%)	1086 (11.4%)	992 (12.2%)	559 (12.1%)
>45 to <50mg	35 (0.4%)	18 (0.2%)	22 (0.3%)	23 (0.3%)	22 (0.2%)	19 (0.2%)	19 (0.2%)	18 (0.2%)	17 (0.4%)
50mg	706 (8.0%)	665 (8.0%)	611 (7.7%)	566 (7.1%)	583 (6.5%)	592 (6.3%)	380 (4.0%)	275 (3.4%)	135 (2.9%)

© 2025 Garg R et al. *JAMA Network Open*.

>50 to <55mg	15 (0.2%)	20 (0.2%)	6 (0.1%)	14 (0.2%)	23 (0.3%)	11 (0.1%)	18 (0.2%)	17 (0.2%)	11 (0.2%)
55mg	288 (3.2%)	255 (3.1%)	194 (2.5%)	214 (2.7%)	212 (2.4%)	261 (2.8%)	241 (2.5%)	190 (2.3%)	147 (3.2%)
>55 to 59mg	14 (0.2%)	7 (0.1%)	1 (0.0%)	14 (0.2%)	18 (0.2%)	12 (0.1%)	11 (0.1%)	13 (0.2%)	5 (0.1%)
60mg	442 (5.0%)	365 (4.4%)	297 (3.8%)	307 (3.8%)	440 (4.9%)	556 (5.9%)	759 (8.0%)	772 (9.5%)	418 (9.0%)
>60 to 100mg	307 (3.5%)	286 (3.5%)	219 (2.8%)	198 (2.5%)	296 (3.3%)	434 (4.6%)	612 (6.4%)	536 (6.6%)	377 (8.2%)
>100mg	146 (1.6%)	91 (1.1%)	67 (0.8%)	60 (0.8%)	50 (0.6%)	52 (0.6%)	66 (0.7%)	94 (1.2%)	61 (1.3%)

© 2025 Garg R et al. *JAMA Network Open*.

eTable 5. Medication Use Characteristics During the First Two Weeks of Treatment, Reported Overall and Stratified by Methadone Dose Dispensed on Index-Date

	Dose <30mg	Dose 30 to <40mg	Dose 40 to <50mg	Dose 50 to <60mg	Overall
**Number of continuous use periods (N)**	38,446	31,870	1,911	1,406	73,633
**Unique Individuals (N)**	24,873	17,474	1,637	1,196	35,309
**Medication(s) dispensed on index-date**					
Combination therapy	180 (0.5%)	2,850 (8.9%)	33 (1.7%)	6 (0.4%)	3,069 (4.2%)
Methadone monotherapy	38,266 (99.5%)	29,020 (91.1%)	1,878 (98.3%)	1,400 (99.6%)	70,564 (95.8%)
Provision of combination therapy within 14 days of index date	124 (0.3%)	514 (1.6%)	22 (1.2%)	7 (0.5%)	667 (0.9%)
**Date of First Dose Increase**					
Index date+1 to index-date+5	18,052 (47.0%)	11,312 (35.5%)	320 (16.7%)	146 (10.4%)	29,830 (40.5%)
Index-date+6 to index-date+13	7,289 (19.0%)	5,722 (18.0%)	252 (13.2%)	154 (11.0%)	13,417 (18.2%)
No dose increase	13,105 (34.1%)	14,836 (46.6%)	1,339 (70.1%)	1,106 (78.7%)	30,386 (41.3%)
**Dose difference between maximum methadone dose dispensed in interval 3 and interval 1**					
No dose dispensed in interval 3	8,895 (23.1%)	9,506 (29.8%)	624 (32.7%)	469 (33.4%)	19,494 (26.5%)
No change	4,998 (13.0%)	5,921 (18.6%)	602 (31.5%)	555 (39.5%)	12,076 (16.4%)
Dose decrease	840 (2.2%)	658 (2.1%)	150 (7.8%)	110 (7.8%)	1,758 (2.4%)
>0 to <15mg	8,939 (23.3%)	3,066 (9.6%)	217 (11.4%)	140 (10.0%)	12,362 (16.8%)
15 to <30mg	9,138 (23.8%)	6,861 (21.5%)	189 (9.9%)	73 (5.2%)	16,261 (22.1%)
30 to <44mg	4,596 (12.0%)	4,208 (13.2%)	90 (4.7%)	29 (2.1%)	8,923 (12.1%)
≥45mg	1,040 (2.7%)	1,650 (5.2%)	39 (2.0%)	30 (2.1%)	2,759 (3.7%)
**Maximum Dose dispensed during treatment days 2-7, N (%)**					
<30mg	19,979 (52.0%)	341 (1.1%)	23 (1.2%)	13 (0.9%)	20,356 (27.6%)
30 to <40mg	11,423 (29.7%)	15,737 (49.4%)	37 (1.9%)	10 (0.7%)	27,207 (36.9%)
40 to <50mg	2,157 (5.6%)	9,827 (30.8%)	975 (51.0%)	18 (1.3%)	12,977 (17.6%)
50 to <60mg	245 (0.6%)	576 (1.8%)	231 (12.1%)	750 (53.3%)	1,802 (2.4%)
≥60mg	415 (1.1%)	1,113 (3.5%)	95 (5.0%)	156 (11.1%)	1,779 (2.4%)
No methadone dispensed in interval 2	4,227 (11.0%)	4,276 (13.4%)	550 (28.8%)	459 (32.6%)	9,512 (12.9%)

© 2025 Garg R et al. *JAMA Network Open*.

**Maximum Dose dispensed during treatment days 8-14, N (%)**					
<30mg	9,363 (24.4%)	608 (1.9%)	25 (1.3%)	21 (1.5%)	10,017 (13.6%)
30 to <40mg	8,725 (22.7%)	6,595 (20.7%)	89 (4.7%)	19 (1.4%)	15,428 (21.0%)
40 to <50mg	6,136 (16.0%)	6,765 (21.2%)	696 (36.4%)	44 (3.1%)	13,641 (18.5%)
50 to <60mg	3,390 (8.8%)	2,504 (7.9%)	233 (12.2%)	618 (44.0%)	6,745 (9.2%)
≥60mg	1,937 (5.0%)	5,892 (18.5%)	244 (12.8%)	235 (16.7%)	8,308 (11.3%)
No methadone dispensed in interval 3	8,895 (23.1%)	9,506 (29.8%)	624 (32.7%)	469 (33.4%)	19,494 (26.5%)

© 2025 Garg R et al. *JAMA Network Open*.

eTable 6. Baseline Characteristics, Reported Overall and Stratified by Methadone Dose Dispensed on Index-Date

	Dose <30mg	Dose 30 to <40mg	Dose 40 to <50mg	Dose 50 to <60mg	Overall
**Number of continuous use periods (N)**	38,446	31,870	1,911	1,406	73,633
**Unique Individuals (N)**	24,873	17,474	1,637	1,196	35,309
**Age, median (IQR)**	33 (27-42)	33 (28-40)	33 (29-40)	34 (29-41)	33 (28-41)
**Age category, N (%)**					
18-24	5,036 (13.1%)	3,210 (10.1%)	177 (9.3%)	124 (8.8%)	8,547 (11.6%)
25-44	25,796 (67.1%)	23,759 (74.5%)	1,425 (74.6%)	1,019 (72.5%)	51,999 (70.6%)
45-64	7,216 (18.8%)	4,784 (15.0%)	290 (15.2%)	247 (17.6%)	12,537 (17.0%)
65+	398 (1.0%)	117 (0.4%)	19 (1.0%)	16 (1.1%)	550 (0.7%)
**Sex, N (%)**					
Female	15,310 (39.8%)	11,930 (37.4%)	688 (36.0%)	493 (35.1%)	28,421 (38.6%)
Male	23,136 (60.2%)	19,940 (62.6%)	1,223 (64.0%)	913 (64.9%)	45,212 (61.4%)
**Region of residence, N (%)**					
Urban	33,333 (86.7%)	27,872 (87.5%)	1,447 (75.7%)	1,058 (75.2%)	63,710 (86.5%)
Rural	4,722 (12.3%)	3,381 (10.6%)	402 (21.0%)	318 (22.6%)	8,823 (12.0%)
Missing	391 (1.0%)	617 (1.9%)	62 (3.2%)	30 (2.1%)	1,100 (1.5%)
**Neighbourhood income quintile, N (%)**					
1 (lowest)	15,353 (39.9%)	13,326 (41.8%)	794 (41.5%)	550 (39.1%)	30,023 (40.8%)
2	8,869 (23.1%)	7,274 (22.8%)	398 (20.8%)	327 (23.3%)	16,868 (22.9%)
3	6,080 (15.8%)	4,972 (15.6%)	260 (13.6%)	209 (14.9%)	11,521 (15.6%)
4	4,334 (11.3%)	3,321 (10.4%)	197 (10.3%)	158 (11.2%)	8,010 (10.9%)
5 (highest)	3,359 (8.7%)	2,331 (7.3%)	200 (10.5%)	131 (9.3%)	6,021 (8.2%)
Missing	451 (1.2%)	646 (2.0%)	62 (3.2%)	31 (2.2%)	1,190 (1.6%)
**Prescribed medications 180 days before index, N (%)**	14,928 (38.8%)	17,832 (56.0%)	1,141 (59.7%)	864 (61.5%)	34,765 (47.2%)
Methadone	12,265 (31.9%)	15,690 (49.2%)	1,069 (55.9%)	829 (59.0%)	29,853 (40.5%)
Buprenorphine/Naloxone	3,378 (8.8%)	3,574 (11.2%)	139 (7.3%)	76 (5.4%)	7,167 (9.7%)

© 2025 Garg R et al. *JAMA Network Open*.

Sublocade	39 (0.1%)	150 (0.5%)	5 - 10	<=5	198 (0.3%)
SROM	311 (0.8%)	2,062 (6.5%)	81 (4.2%)	22 (1.6%)	2,476 (3.4%)
Kadian	280 (0.7%)	2,042 (6.4%)	81 (4.2%)	21 (1.5%)	2,424 (3.3%)
M-ESLON	34 (0.1%)	34 (0.1%)	<=5	<=5	70 (0.1%)
Immediate Release Hydromorphone	695 (1.8%)	412 (1.3%)	21 (1.1%)	15 (1.1%)	1,143 (1.6%)
**Occurrence of opioid toxicity in 365-days prior to index date, N (%)**	3,169 (8.2%)	3,998 (12.5%)	210 (11.0%)	120 (8.5%)	7,497 (10.2%)
**Prescriber Type and Specialty, N (%)**					
Physician	38,194 (99.3%)	31,468 (98.7%)	1,881 (98.4%)	1,391 (98.9%)	72,934 (99.1%)
Family Practitioner	30,216 (78.6%)	23,789 (74.6%)	1,409 (73.7%)	1,056 (75.1%)	56,470 (76.7%)
Emergency Medicine	4,243 (11.0%)	3,120 (9.8%)	230 (12.0%)	171 (12.2%)	7,764 (10.5%)
Internal Medicine	36 (0.1%)	19 (0.1%)	<=5	<=5	62 (0.1%)
Psychiatry	1,971 (5.1%)	1,772 (5.6%)	91 (4.8%)	69 (4.9%)	3,903 (5.3%)
Other	1,980 (5.2%)	3,170 (9.9%)	179 (9.4%)	105 (7.5%)	5,434 (7.4%)
Nurse	252 (0.7%)	402 (1.3%)	30 (1.6%)	15 (1.1%)	699 (0.9%)
**OAT prescribing volume**					
Low (50th percentile)	1,380 (3.6%)	1,080 (3.4%)	169 (8.8%)	129 (9.2%)	2,758 (3.7%)
Moderate (51st to 80th percentile)	9,935 (25.8%)	7,693 (24.1%)	469 (24.5%)	362 (25.7%)	18,459 (25.1%)
High (top 20th percentile)	27,131 (70.6%)	23,097 (72.5%)	1,273 (66.6%)	915 (65.1%)	52,416 (71.2%)

© 2025 Garg R et al. *JAMA Network Open*.

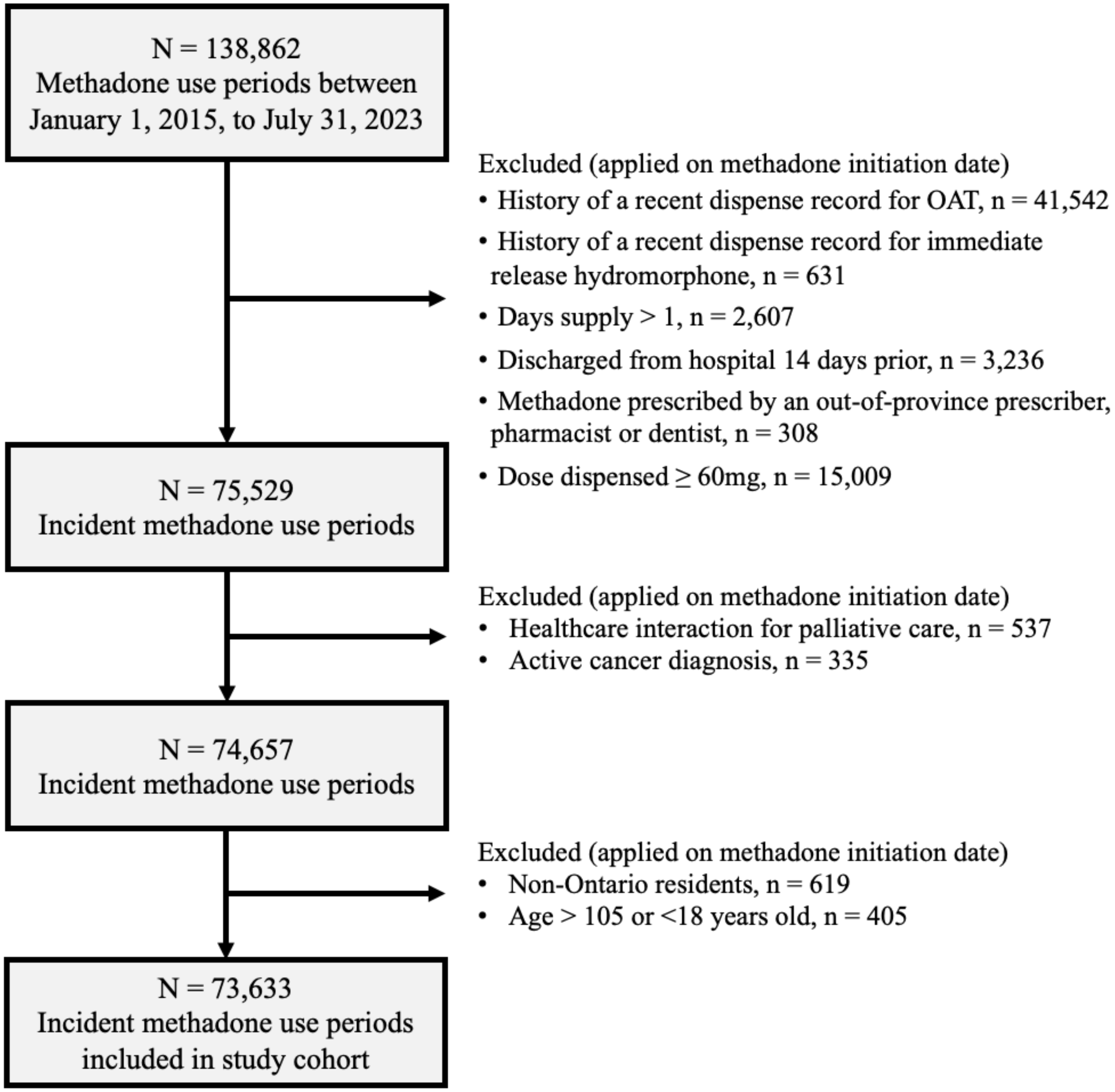

eFigure 1. Cohort Exclusion Flow Diagram.

© 2025 Garg R et al. *JAMA Network Open*.

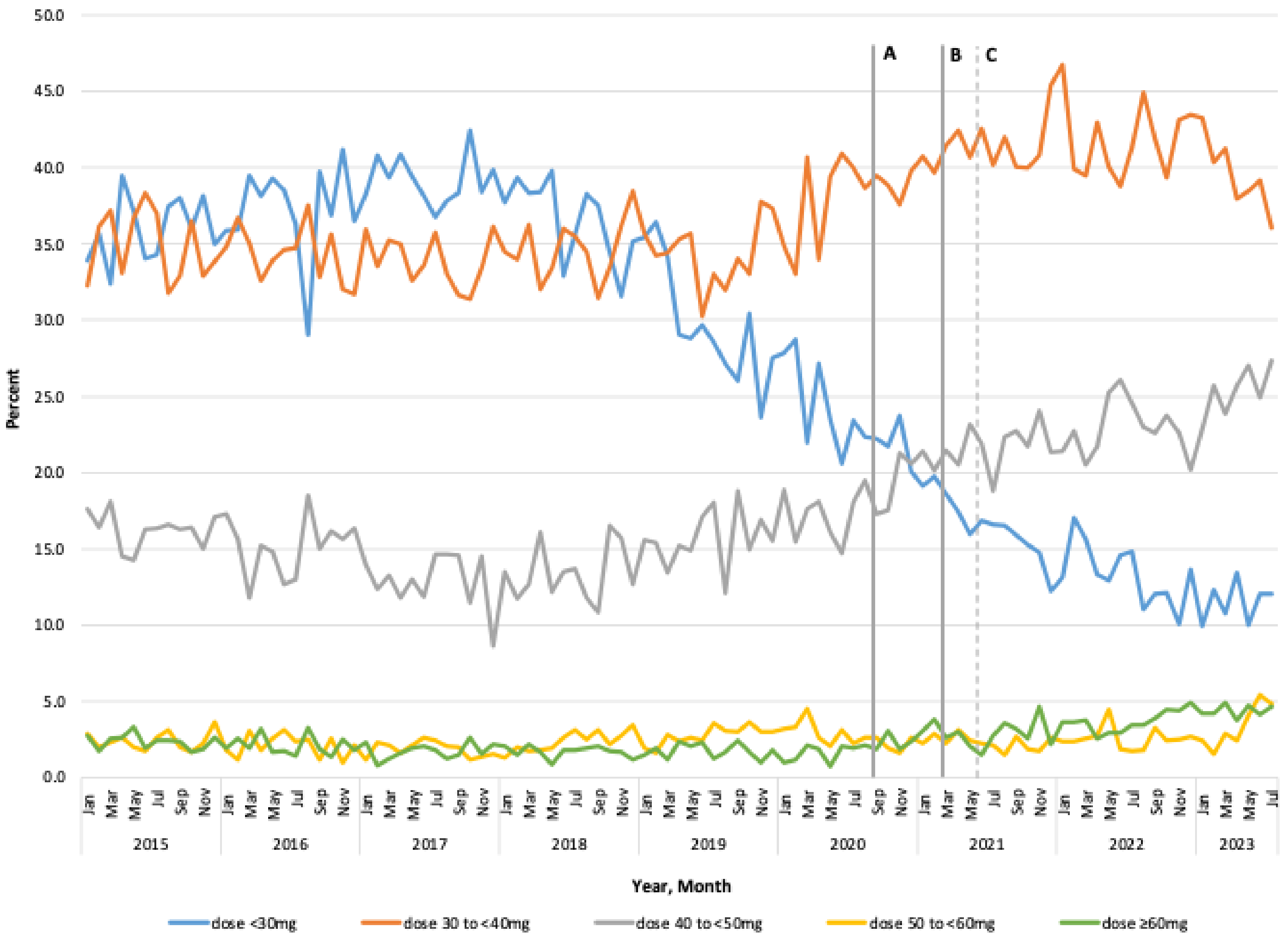

eFigure 2. Maximum Methadone Dose Dispensed Between the Second to Seventh Day of Treatment Among Incident Methadone Recipients in Ontario, Canada, Between January 2015 to July 2023

Notes: Vertical bars represent months of overlapping dates, with solid bars representing intervention months included in autoregressive interventional moving average models: A = META-PHI Annual Conference (September 2020); B = Release of preliminary methadone prescribing recommendations by META-PHI and rescindment of CPSO guidelines; and C = Official release of methadone prescribing recommendations by META-PHI.

© 2025 Garg R et al. *JAMA Network Open*.

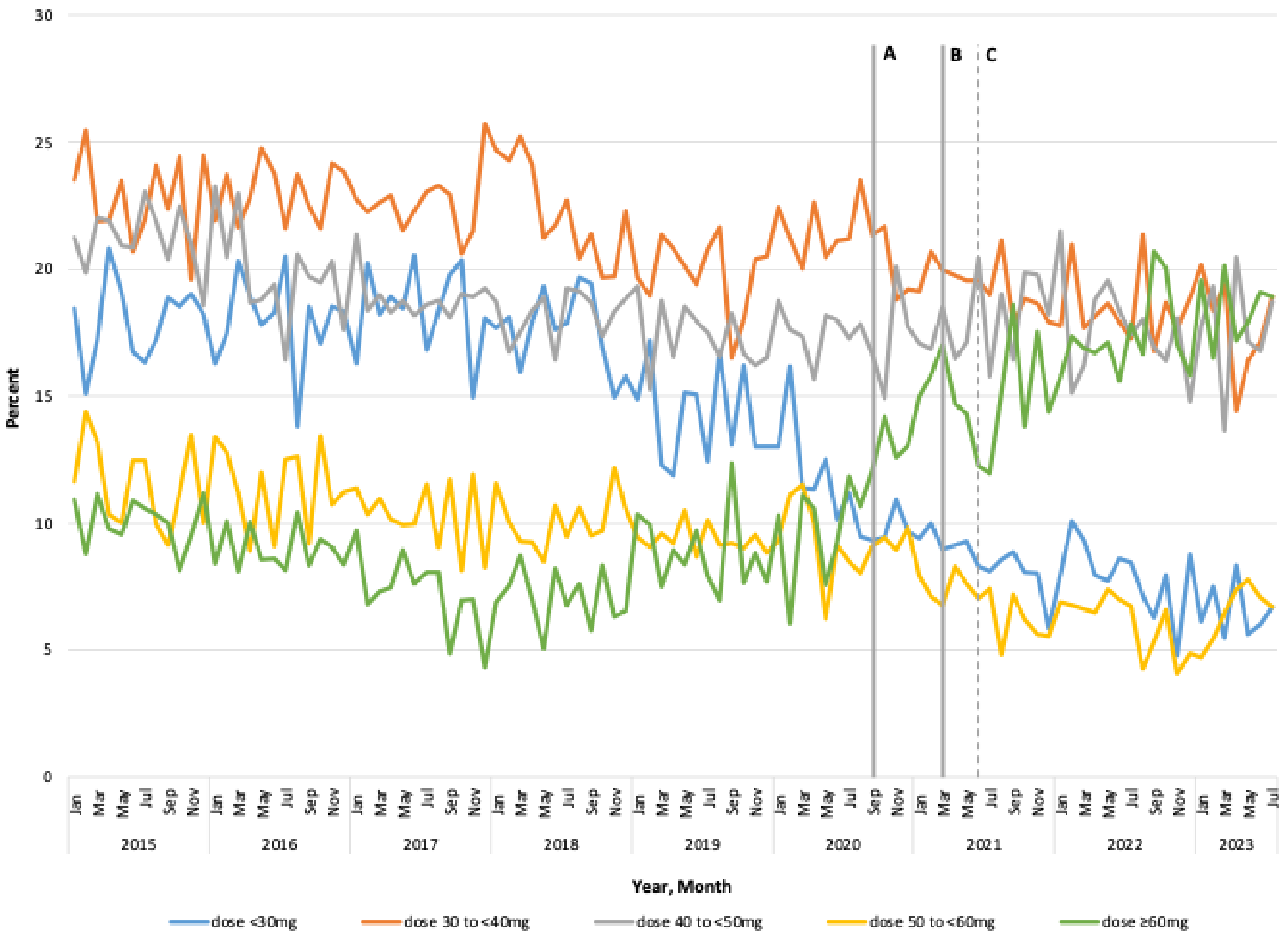

eFigure 3. Maximum Methadone Dose Dispensed Between the Eighth to 14th Day of Treatment Among Incident Methadone Recipients in Ontario, Canada, Between January 2015 to July 2023

Notes: Vertical bars represent months of overlapping dates, with solid bars representing intervention months included in autoregressive interventional moving average models: A = META-PHI Annual Conference (September 2020); B = Release of preliminary methadone prescribing recommendations by META-PHI and rescindment of CPSO guidelines; and C = Official release of methadone prescribing recommendations by META-PHI.

© 2025 Garg R et al. *JAMA Network Open*.
