# Supplementary material for: Initiation and Dose of Methadone Monotherapy vs Combination Therapy, 2015 to 2023
Source: JAMA Netw Open. 2025 Aug 15;8(8):e2527290. doi: 10.1001/jamanetworkopen.2025.27290 (PMC12357187; doi:10.1001/jamanetworkopen.2025.27290)
Supplement: Supplement 2. — Data Sharing Statement [file jamanetwopen-e2527290-s002.pdf]

Data Sharing Statement

Garg. Initiation and Dose of Methadone Monotherapy vs Combination Therapy, 2015 to 2023. *JAMA Netw Open*. Published August 15, 2025. doi:10.1001/jamanetworkopen.2025.27290

Data
Data available: No
